# Ethnic delineation of primary glioblastoma genome

**DOI:** 10.1002/cam4.3370

**Published:** 2020-08-13

**Authors:** Harim Koo, Seung Won Choi, Hee Jin Cho, In‐Hee Lee, Doo‐Sik Kong, Ho Jun Seol, Jung‐Il Lee, Jung‐Won Choi, Jason K. Sa, Do‐Hyun Nam

**Affiliations:** ^1^ Department of Health Sciences and Technology SAIHST Sungkyunkwan University Seoul Republic of Korea; ^2^ Department of Clinical Research Research Institute and Hospital National Cancer Center Goyang Republic of Korea; ^3^ Department of Neurosurgery Sungkyunkwan University School of Medicine Samsung Medical Center Seoul Republic of Korea; ^4^ Innovative Therapeutic Research Center Precision Medicine Research Institute Samsung Medical Center Seoul Republic of Korea; ^5^ Computational Health Informatics Program Boston Children’s Hospital Boston MA USA; ^6^ Department of Biomedical Sciences Korea University College of Medicine Seoul Republic of Korea

**Keywords:** ethnic, genetics, genomics, glioblastoma

## Abstract

Glioblastoma (GBM) is the most malignant primary brain tumor in adults with substantial genomic alterations. The median survival is approximately 14.6 months, despite aggressive therapeutic intervention, which comprised of surgical resection, radiotherapy, and chemotherapy. Recent studies on cancer genomic have revealed crucial insights into dynamic molecular subgroups within GBM, which govern distinct clinical response and sensitivity of each individual to therapy. In the present study, we analyzed genomic composition of primary GBMs between two ethnic groups [IRCR (Institute of Refractory Cancer Research), and TCGA (The Cancer Genome Atlats)] to explore genomic and molecular features that constitute malignant behavior of glioblastoma based on distinct ethnicity. We identified enrichments of MAPK and p53 pathways in IRCR patients, while aberrant activation of Receptor Tyrosine Kinases (RTKs) were predominant in TCGA cohort. We also discovered differential clinical prognosis between two groups and explored essential features that present such diversity.

## Graphical abstract

Glioblastoma (GBM) is the most aggressive primary brain tumor. We analyzed recurrent somatic variants and molecular pathways that are enriched in different subpopulations.

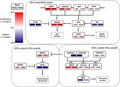

## 1 INTRODUCTION

Glioblastoma (GBM) is the most common and malignant primary brain tumor in adults with profound genetic alterations.^1^, ^2^, ^3^ Current standard therapeutic regimen, which comprised of surgical resection followed by radiotherapy and chemotherapy, provides only palliation with 5‐year survival rate of less than 10%.^1^, ^2^ GBM is a complex disease with extensive intra‐ and inter‐tumoral heterogeneity, highlighting distinct molecular and epigenetic states that dictate clinical prognosis and sensitivity of individual patient to particular therapy.^4^, ^5^, ^6^, ^7^, ^8^ As substantial number of studies have recently adopted high‐throughput sequencing technology, large‐scale genomic analyses have provided unprecedented insights into complex genomic and molecular underpinnings of GBM progression.^4^, ^8^, ^9^, ^10^ Notably, The Cancer Genome Atlas Consortium (TCGA) have identified molecular subclasses within GBM and core molecular pathways, including receptor tyrosine kinase (RTK)/Ras/phosphoinositide 3‐kinase (PI3K), Rb, and p53 that are frequently dysregulated.^2^, ^4^, ^9^, ^10^, ^11^, ^12^

Although genome based glioma classification has been well‐established, it is solely based on TCGA dataset, which mainly consists of non‐Asian populations (82% Caucasians, 2% Hispanics, etc).^4^, ^9^ As recent studies have highlighted ethnic delineation of differential genetic pathways across multiple cancer lineages,^9^ evaluation of population differences in genetic susceptibility may provide unprecedented insights into alternative molecular pathways that are actively enriched. As there is currently no effective treatment option that is readily available for patients with recurrent glioblastomas, identification differential molecular pathways that are explicit to specific population could facilitate exploration of new therapeutic approach in an event of tumor relapse.

Toward this goal, we have characterized genomic profiles and molecular pathways that are implicated in gliomagenesis from 90 Korean patients (IRCR) who were diagnosed with de novo glioblastoma (primary GBMs). We have also evaluated clinical prognosis, genetic alteration frequency of major glioma‐driver genes, and landscape of core molecular signaling pathways between our cohort with TCGA using whole‐exome and whole‐transcriptome sequencing.

## 2 METHOD

### 2.1 Patients and specimens

Surgical specimens and corresponding clinical records were obtained from patients who were diagnosed with glioblastoma and underwent brain tumor removal surgery at Samsung Medical Center (No. 2005‐05‐001, 2010‐04‐004) and The Cancer Genome Atlas. Informed consent was obtained from each patient prior to the start of the study. For genome sequencing, parts of the brain tumor were obtained from surgical resection and snap‐frozen and stored in liquid nitrogen. Whole‐Exome Sequencing and Whole‐Transcriptome Sequencing data were acquired for 250 and 90 TCGA and IRCR cases respectively. This research was designed to specifically evaluate frequency of major driver genetic alterations in glioblastoma based on ethnicity difference in response to standard treatments rather than discover novel variants.

### 2.2 WES

#### 2.2.1 Raw data

2 × 101 base pair paired‐end reads were generated using Illumina HiSeq2000 for genome sequencing.

#### 2.2.2 Somatic mutation

The sequenced reads from the FASTQ files were mapped and aligned to the human genome assembly (hg19) using Burrows‐Wheeler Aligner (Liu et al, 2009). After conventional preprocessing of the initial aligned BAM file, we generated mutation calls. BAM files were preprocessed for sorting, removing of duplicate reads, realigning reads around potential small indels using Picard and GATK, respectively. Furthermore, we used SAMtools to generate and evaluate realignment and recalibrating base quality score. Confidence level for the somatic mutation calls using tumor and matched blood was predicted using MuTect and SomaticIndelDetector (Banerji et al, 2012). Variant Effect Predictor (VEP) was used to annotate somatic mutations with potential functional implications and other significant information.

#### 2.2.3 Copy number

For copy number analysis, We used version 0.4.4 of the ngCGH python package to generate aCGH‐like data from the WES data. Tumors and matched normal blood were used to generate gene‐based read counts. Normalized copy number values were calculated using log2 scale.

#### 2.2.4 RNA‐seq

RNA sequencing libraries were generated using the Illumina TruSeq RNA sample Library Preparation Kit. RNA‐seq data were used to evaluate mRNA expression level, point mutations, and structure variations, including exon skipping and gene fusions. The sequenced reads from the FASTQ files were mapped onto hg19 using GSNAP, preventing potential splicing, indels, or mismatch. As a result, the alignment SAM files were sorted and summarized into BED files using SAMtools and bedTools. The normalized gene expressions were calculated and quantified in Reads Per Kilobase of transcript per Million mapped (RPKM) format. We used the R package DEGseq and RefSeq gene annotations for further process. To identify expression‐based subtypes, the normalized expression data was applied to single‐sample gene set enrichment analysis (ssGSEA) to calculate enrichment scores (ES) for each GBM subclass that were previously defined by Verhaak et al. The representative subtype of for each tumor case was used by applying the highest ES.

## 3 RESULTS

### 3.1 Demographic backgrounds of primary glioblastoma patients in TCGA and IRCR cohorts

To compare the demographic backgrounds of patients with de novo glioblastoma in TCGA and IRCR cohorts, we only included the primary glioblastoma patients without prior treatment history. Thirteen patients in TCGA cohorts were excluded as they had prior treatment history or their treatment histories were not available. 90 primary glioblastoma patients in IRCR cohorts were selected for availability in WES data. Two patients were excluded from the IRCR cohort due to different ethnic origin. As a result, 250 and 90 glioblastoma patients were selected to compare the demographic landscape of Caucasian and Korean patients respectively.

Median age at diagnosis between two cohorts were at 61.4 ± 12.6 and 54.7 ± 11.6 for TCGA and IRCR cohorts respectively. Male patients were dominant in both groups and the male to female ratios were similar [36.8%: 63.2% (TCGA) vs 46.2%: 53.8% (IRCR)]. *IDH1* mutation was rarely detected in both groups and the incidence rate was at 4.4%‐4.8% which was comparable to previous literatures (NEJM, 2009, Yan H et al (5%)/Acta Neuropathol, 2008, Balss J et al (7%)).^13^, ^14^ Demographics and treatment histories are summarized in Table 1.

**TABLE 1:** Demographic backgrounds of TCGA vs IRCR in primary GBM

	TCGA (N = 250)	IRCR (N = 90)	*P*‐value
Gender			
Female	92 (36.8%)	41 (45.6%)	.15
Male	158 (63.2%)	49 (54.4%)	
Age	61.4 ± 12.6	54.7 ± 11.6	.001
IDH1 (250 vs 90)			
MUTANT	12 (4.8%)	4 (4.4%)	1.000
WT	238 (95.2%)	86 (95.6%)	
Treatment (234 vs 88)			
Stupp regimen	95 (40.6%)	87 (98.9%)	2.2e‐16
Radiation therapy	228 (97.4%)	88 (100%)	.1404
Chemotherapy	162 (69.2%)	87 (98.9%)	
TMZ chemo	154 (65.8%)	87 (98.9%)	
Stupp regimen completion	Unknown	46 (52.3%)	

^a^ Student *t*‐test was used for continuous variables and fisher's exact test was used to compare the categorical variables. *P*‐values below .05 were set to be statistically significant.

**FIGURE 1:**
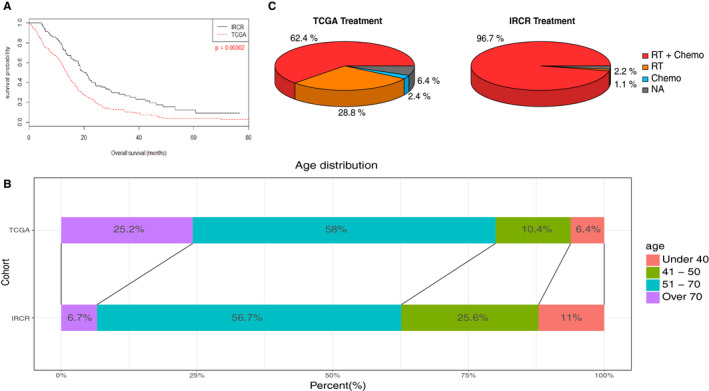
Overall survival of primary glioblastoma patients in TCGA and IRCR cohorts. To compare the survival outcome of primary glioblastoma patients between two cohorts, Kaplan‐meier survival curves were plotted. Survival outcome was significantly worse in TCGA cohorts (*P* = .00002 (Log‐rank test), median overall survival, 11.8 months vs 19.1 months). Younger patients (age < 50 years) were more prevalent in IRCR cohorts while older patients (age > 70 years) comprised up to one‐fourth of total population in TCGA cohorts (Figure 1B). Treatment strategy was significantly different between two cohorts; IRCR patients significantly had more temozolomide and chemotherapy other than temozolomide compared to TCGA cohorts (Figure 1C). Most of IRCR patients undertook the Stupp regimen and half of them completed the standard protocol while only 40% of total population had the Stupp regimen in TCGA cohorts (Table 1)

Survival outcome was significantly worse in TCGA cohort (*P* < .001, Log‐rank test, Figure 1A). Median overall survival was 11.85 months in TCGA cohort while 19.1 months in IRCR cohort. To elucidate the survival difference between two groups, we examined age distribution between two cohorts (Figure 1B). Younger patients [age < 50 years, 16.8% (TCGA) vs 36.6% (IRCR)] were more prevalent in IRCR cohort, while older patients comprised one‐fourth of TCGA cohort [age > 70, 25.2% (TCGA) vs 6.7% (IRCR)]. As age is considered as an essential prognostic factors in glioblastoma patients,^15^ survival difference between two cohorts were suspected to be derived from distinct age distributions. Another potential contributing factor for the survival difference was due to different therapeutic strategy (Figure 1C). Most of IRCR patients had undergone surgery after year 2008; thus, majority of them followed the Stupp regimen while only a subset of TCGA patients adopted the same protocol.^16^ When we stratified the patients according to differential treatment strategy, survival gain on Stupp regimen was more significant (Figure S1).

### 3.2 The genomic landscape of primary glioblastoma in TCGA and IRCR cohorts

**FIGURE 2:**
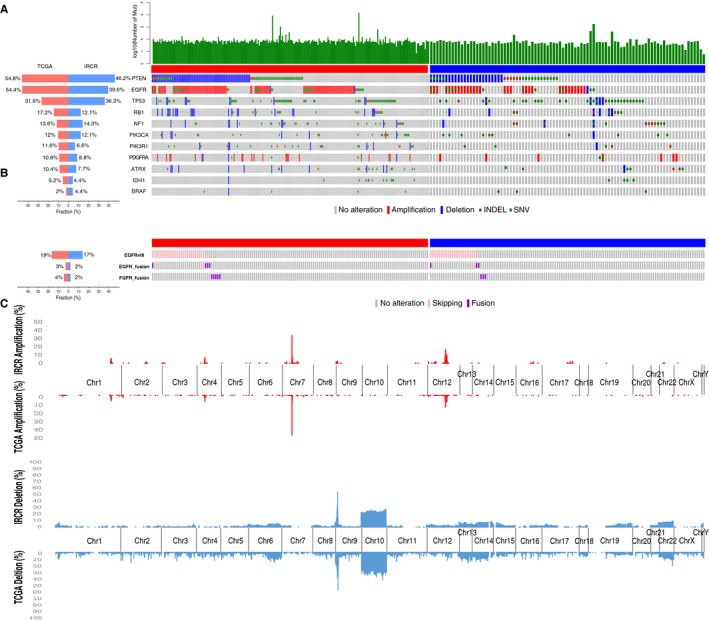
Genomic landscape of primary glioblastoma in TCGA and IRCR cohorts. (A) Each samples in TCGA and IRCR cohorts are annotated for their mutation, copy number alteration, exon skip and fusion. (B) *EGFRvIII*, *EGFR*,and *FGFR* fusions that were detected from two cohorts (136/126 in TCGA/IRCR) with RNA sequencing data are shown. (C) Focally amplification (log2(CN/2) > 1.58, red) and deletion (log2(CN/2) < −0.75, blue) are plotted on each chromosome. Bottom side: TCGA, Top side: IRCR

We obtained Whole‐Exome Sequencing (WES) data for both tumors and matched blood and the authenticity of somatic mutations are clearly apparent in these cases. For somatic mutation analysis, we employed MuTect, a widely used software for generating confident mutation calls.^17^ Both TCGA and IRCR datasets included WES and Whole‐Transcriptome Sequencing (WTS). To account for different methodological and analytical approach in acquiring somatic mutation and copy number variation calls, we have processed both IRCR and TCGA cohorts using the same pipeline, which consists of SAMtools and Genome Analysis Toolkit (GATK) version 2.5.2 for genome alignment, Picard version 1.73 for removal of read duplication etc. As summarized in Figure 2A, both cohorts harbored multiple somatic genomic aberrations in the major oncogenic pathways that are frequently dysregulated in glioblastoma, including receptor tyrosine kinase (RTK), p53, and Phosphoinositide 3‐kinase (PI3K) signaling pathways.^18^, ^19^, ^20^, ^21^, ^22^, ^23^ The most prevalent genomic alterations consisted of *PTEN* (55%/46% in TCGA/IRCR), *EGFR* (54%/40% in TCGA/IRCR), and *TP53* (31%/36% in TCGA/IRCR), demonstrating similar frequencies between two cohorts. *ATRX* and *IDH1* mutations appeared to be more commonly altered in glioblastoma that lack RTK genomic variations, genetically resembling to that of secondary GBM.^12^ There were four cases with more than 500 mutated genes (Hypermutation) in TCGA cohort and one sample in IRCR cohort. For copy number variations, genomic amplification was marked as harboring more than six copies, while deletion was labeled as less than 1.2 copy. *EGFR* amplification and *PTEN* deletion were more prevalent in TCGA (49.6%/35.6%, respectively) compared to IRCR cohort (32%/26.3%), suggesting that IRCR tumors may undergo alternative molecular pathways during tumor propagation. In contrast, genetic alterations in *TP53*, *RB1*, and *NF1* genes were observed at a similar fraction. 136 and 126 tumors were available for Whole‐Transcriptome Sequencing for TCGA and IRCR respectively. Structure variations in *EGFR*, including 2‐7 exon deletion (*EGFRvIII*) and genomic‐fusions (*EGFR*, *FGFR*), were observed at 19%/3%/4% in TCGA and 17%/2%/2% for IRCR cohorts (Figure 2B).^18^, ^24^ We observed several recurrent chromosomal aberrations that are often dysregulated in glioblastoma progression in both cohorts including amplification of chromosome 7 and 4, harboring *EGFR* and *PDGFRA* respectively.^22^ We also detected copy number co‐amplification of *CDK4* and *MDM2* in chromosome 12. Furthermore, both cohorts exhibited significant deletion in chromosome 10, one of the major genomic features of glioblastoma, and chromosome 9, harboring *CDKN2A* and *CDKN2B* (Figure 2C).

### 3.3 Molecular subtypes of glioblastoma

As previous studies have unveiled presence of distinct GBM subtypes based on their unique genomic alterations and transcriptome expression profiles,^5^, ^23^, ^25^ we have evaluated classification frequency of both conventional and tumor‐intrinsic GBM subtypes in both cohorts.

The new classification of tumor‐intrinsic subtypes were configured with 50 genes to generate single sample gene set enrichment analysis (ssGSEA) based activity score. Each subtypes is accompanied with unique genomic alterations including genomic alterations of *EGFR*, *NF1*, and *PDGFRA/IDH1* for classical (CL), Mesenchymal (MES), and Proneural (PN), respectively. Neural (NL) did not exhibit any distinguishable genomic variants as it showed a strong relationship with the gene expression signatures of normal brain cells. Furthermore, each subtype demonstrated distinct therapeutic response as CL tumors benefitted the most from the standard treatment, while PN exhibited the least response. Additionally, each subtype showed differential chromosomal aberrations as Classical subtype showed recurrent copy number amplification of chr7 with loss of chr10, whereas Mesenchymal tumors were marked by focal hemizygous deletion 17q11.2.^5^, ^22^, ^25^, ^26^, ^27^, ^28^

**FIGURE 3:**
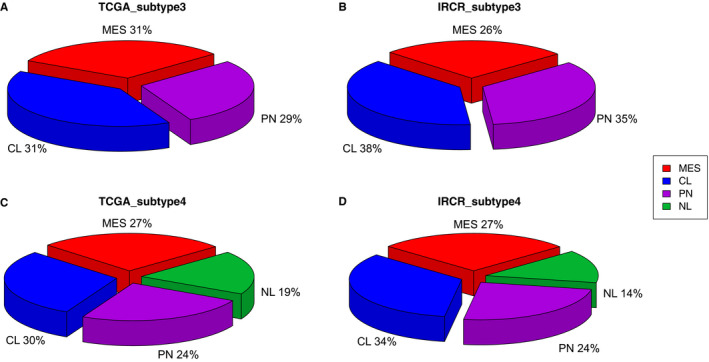
Molecular subtypes of primary glioblastoma in TCGA and IRCR cohorts. MES, Mesenchymal; CL, Classical; PN, Proneural; NL, Neural

136 and 126 mRNA sequenced data from TCGA and IRCR cohorts were enrolled to evaluate subtype classification analysis. In TCGA group, MES subtype proportions were measured at 31%(43/136), PN subtype proportions at 29%(40/136), and CL subtype proportions at 40%(55/136) (Figure 3A), while MES subtype proportions were observed at 26%(33/126), PN subtype proportions at 35%(44/126), and CL subtype proportions at 39%(49/126) in IRCR cohort (Figure 3B). For four‐subtype cluster, the same analytical method has been applied. In TCGA, MES subtype proportions were found in 27%(37/136), PN subtype proportions in 24%(34/136), CL subtype proportions in 30%(41/136), and NL subtype proportions in 19% (26/136) (Figure 3C). In IRCR, MES subtype proportions were shown at 27%(34/126), PN subtype proportions at 25%(31/126), CL subtype proportions at 34%(43/126), and NL subtype proportions at 14% (18/126) (Figure 3D).

### 3.4 Landscape of pathway alterations in primary GBM

**FIGURE 4:**
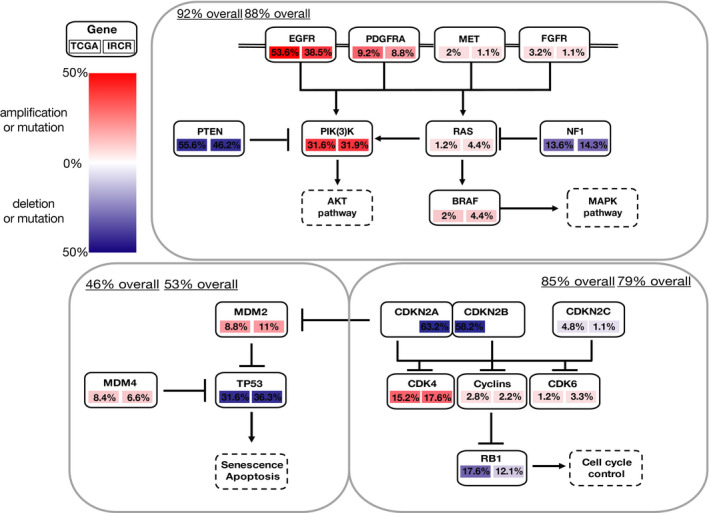
Landscape of major pathway alterations in primary GBM. Overall analysis proportion is summarized for PI3K/MAPK, p53 and Rb regulatory pathway. Amplification (log2(CN/2) > 1.58) and deletion (log2(CN/2) < −0.75, blue)

Glioblastoma genome is composed of three major molecular canonical pathways, including RTK/PI3K/MAPK, p53, and Rb regulatory pathways. By combining mutation profiles with copy number variations using whole‐exome sequenced data, we have evaluated which particular oncogenic pathway was more actively enriched based on ethnic difference. Among many genomic alterations, we have closely interrogated genes that were involved in the p53 pathway (*MDM4*, *MDM2*, and *TP53*), the Rb pathway (*CCND2*, *CDKN2A/B*, *CDK4*, *CDK6*, and *RB1*), and the RTK/PI3K/MAPK pathway (*PIK3CA*, *PIK3R1*, *EGFR*, *PTEN*, *PDGFRA*, *BRAF* and *NF1*).^4^ As previously discussed, major core oncogenic pathways were found to be dysregulated in large fractions of primary GBM.^9^ In both TCGA and IRCR cohorts, major genomic alteration events consist of mutation and copy number alterations of RTK/PI3K/MAPK pathways. *EGFR* alterations were the most prominent events in both cohorts (53.6%/38.5%), followed by PI3K signaling pathway (composed of *PTEN* mutation/deletion and *PIK3CA* mutation) (72.8%/67%). Notably, TCGA cohort showed more prevalent dysregulation of RTKs including *EGFR, PDGFRA* (9.2%/8.8%)*, MET* (2%/1.1%), and *FGFR* (3.2%/1.1%). On the contrary, activation of MAPK pathway was more frequent in IRCR cohort, with mutations in *BRAF* (2%/4.4%), which has been previously reported to be associated with gliomagenesis, and chromosomal deletion and mutation of *NF1* (11.6%/14.3%). Furthermore, IRCR cohorts showed enrichment of p53 pathway, which consists of deletion and mutation in *TP53* (31.6%/36.3%), and genomic amplification of *MDM2/4* (17.2%/17.7%). Lastly, we observed dysregulation of Rb functional pathway in both cohorts, which consists *RB1* mutation and deletion (17.6%/12.1%), *CDK4/6* amplification (16.4%/20.9%), and genomic deletion of *CDKN2A/B* (63.2%/58.2%) (Figure 4). Collectively, our results explore dynamic activation of core oncogenic pathways that modulate tumor progression and potentially dictate therapeutic response to distinct therapeutic approach.

## 4 DISCUSSION

Molecular and genomic characterization of tumors enables identification of effective drugs tailored to individual patients. Therefore, exploration and interpretation of cancer genomes are essential in extending the survival benefits in patients with GBM. As there is are huge limitations on effective treatment strategies that are readily available for patients with recurrent GBMs, identification of specific molecular targets or pathways could facilitate exploration of alternative therapeutic approach. Previous studies have demonstrated aberrant activation of major oncogenic pathways that are frequently altered in glioblastoma, making them ideal therapeutic targets within clinical framework. However, as ethnic delineation largely constitutes distinct tumor genomic and molecular compositions across multiple cancer types, exploration of ethnic‐based molecular pathway has been necessary in designing ideal therapeutic strategy for each subpopulations. Previous study has demonstrated influence of genetic ancestry on genomic alterations in a broad range of different tumor types. For example, *TP53* mutations and *CCNE1* genomic amplifications were more prevalent in African Americans compared to European Americans. Furthermore, recurrent focal amplification within the chromosomal 19q12 region was significantly more frequent in multiple solid tumors, including GBM.^29^ In such context, our study presents new opportunities to explore the impact of genetic ancestry in GBM genome in Asian populations through direct comparison.

Toward this goal, we have evaluated genomic and transcriptomic landscape of IRCR (Korean) patients with TCGA cohort, which mainly consist of Non‐Asian populations. Although, both groups exhibited similar genomic profiles, including chromosomal amplification and deletion of 7 and 10, respectively, they demonstrated enrichment of distinct core molecular pathways that could potentially dictate diverse pharmacological response to targeted therapies. IRCR patients showed activation of MAPK and p53 pathways, while genetic aberration of RTKs were more prominent in TCGA cohort. Our results suggest potential benefits of MAPK or p53 inhibitors for Korean GBM patients.

Furthermore, we have discovered a significant survival difference between IRCR and TCGA cohorts, which could be derived from distinct age distribution among the patients. As IRCR patients are diagnosed at a much earlier age compared to TCGA cohort, further exploration on differential age distribution gap could provide unprecedented insights into molecular determinants that dictate gliomagenesis. Collectively, our results highlight genetic heterogeneity in glioblastoma based on distinct ethnicity and its significance in designing specific therapeutic strategy for each subgroups.

## CONFLICTS OF INTEREST

The authors have no potential conflict of interests to disclose.

## AUTHOR CONTRIBUTIONS

Harim Koo and Seung Won Choi are co‐first author: conceptualization, validation, data curation, and writing and editing of the original draft; Hee Jin Cho and In‐hee Lee: validation, formal analysis, and data curation; Doo‐Sik Kong, Ho Jun Seol, Jung‐Il Lee, and Jung‐Won Choi: collection of patient samples, and clinical information; Jason K. Sa and Do‐Hyun Nam: editing, conceptualization, funding, and supervising the entire project.

## Supporting information

Fig S1
